# Diagnostic accuracy and safety of CT-guided percutaneous lung biopsy with a coaxial cutting needle for the diagnosis of lung cancer in patients with UIP pattern

**DOI:** 10.1038/s41598-022-20030-z

**Published:** 2022-09-20

**Authors:** Da Young Kim, Joo Sung Sun, Eun Young Kim, Kyung Joo Park, Seulgi You

**Affiliations:** 1 Department of Radiology, Ajou University School of Medicine, 164 Worldcup Road, Yeongtong-gu, Suwon, 16499 Republic of Korea; 2 Department of Radiology, Hankook Hospital, Cheongju, Republic of Korea

**Keywords:** Laboratory techniques and procedures, Tomography, Lung cancer

## Abstract

This study aimed to assess the diagnostic accuracy and safety of CT-guided percutaneous core needle biopsy (PCNB) with a coaxial needle for the diagnosis of lung cancer in patients with an usual interstitial pneumonia (UIP) pattern of interstitial lung disease. This study included 70 patients with UIP and suspected to have lung cancer. CT-guided PCNB was performed using a 20-gauge coaxial cutting needle. The diagnostic accuracy, sensitivity, specificity, and percentage of nondiagnostic results for PCNB were determined in comparison with the final diagnosis. PCNB-related complications were evaluated. Additionally, the risk factors for nondiagnostic results and pneumothorax were analyzed. The overall diagnostic accuracy, sensitivity, and specificity were 85.7%, 85.5%, and 87.5%, respectively. The percentage of nondiagnostic results was 18.6% (13/70). Two or less biopsy sampling was a risk factor for nondiagnostic results (*p* = 0.003). The overall complication rate was 35.7% (25/70), and pneumothorax developed in 22 patients (31.4%). A long transpulmonary needle path was a risk factor for the development of pneumothorax (*p* = 0.007). CT-guided PCNB using a coaxial needle is an effective method with reasonable accuracy and an acceptable complication rate for the diagnosis of lung cancer, even in patients with UIP.

## Introduction

Usual interstitial pneumonia (UIP) is a histological and radiological pattern of interstitial lung disease (ILD). Although a UIP pattern is a hallmark of idiopathic pulmonary fibrosis (IPF), it can appear not only in IPF but also in other ILDs (e.g., ILD caused by collagen vascular disease, hypersensitivity pneumonitis, and asbestosis)^1^. The prognosis of IPF is notably poor, and the overall median survival has been estimated as 3 years. Also, in patients with fibrosing ILDs other than IPF, UIP pattern is associated with more rapid disease progression and poorer prognosis^2–5^. This condition is also reportedly associated with a high risk of lung cancer; the incidence of lung cancer among patients with IPF is approximately 22.9 per 10,000 person-years^6^. Furthermore, patients with both lung cancer and IPF have poor prognosis and difficulty in proper management due to surgical morbidity and respiratory complication such as drug induced pneumonitis and acute exacerbation of IPF^7,8^. Patients with IPF regularly undergo chest CT for evaluation of disease progression and for the risk of developing lung cancer. In patients suspected of having lung cancer, histological confirmation is needed for accurate diagnosis and proper management.

Percutaneous CT-guided lung biopsy is a well-established technique for the diagnosis of lung cancer with a high diagnostic accuracy (83%–97%) and an acceptable complication rate (22%–51%)^9–15^. However, CT-guided lung biopsy in patients with UIP is challenging because of the patients’ impaired pulmonary function and high risk of pneumothorax^16^. Owing to the underlying lung fibrosis in patients with UIP, re-expansion of the lung is limited and may require chest tube drainage more frequently and for longer duration than for patients without UIP^16^. A recent study evaluated the diagnostic accuracy of CT-guided percutaneous lung biopsy (fine-needle aspiration and core needle biopsy) in patients with a UIP/IPF pattern. The authors reported that CT-guided percutaneous lung biopsy had a reasonable accuracy, but showed a relatively high complication rate (51%) in patients with a UIP/IPF pattern^17^.

Compared with fine-needle aspiration, percutaneous core needle biopsy (PCNB) achieves a comparably high diagnostic accuracy for the diagnosis of lung cancer^18^. In addition, several studies have reported that using a coaxial cutting needle could improve diagnostic accuracy^15,19^ and reduce the pneumothorax rate^20^. As the use of a coaxial needle can avoid repeated traversal of the pleura, a coaxial needle would be a better choice in patients with IPF who are at high risk for pneumothorax. However, no previous study has described the diagnostic performance of CT-guided PCNB using a coaxial cutting needle in patients with UIP.

Thus, the purpose of our study was to assess the diagnostic accuracy and safety of CT-guided PCNB in patients with a fibrosing ILD with a UIP pattern by using a coaxial cutting needle system and to identify the predictive factors for nondiagnostic results and pneumothorax.

## Methods

All procedures were performed in accordance with the relevant guidelines and regulations^14,21^. This clinical observational study was approved by our institutional ethics committee and the need for informed consent was waived (Ajou institutional review board).

### Study design and patients

A total of 1,122 consecutive CT-guided PCNBs for the diagnosis of lung cancer were performed between April 2016 and May 2021 at a tertiary referral hospital. Two board-certified radiologists (19 and 3 years of experience in chest radiology) reviewed all diagnostic chest CT images taken before PCNBs and evaluated the presence of ILD. The diagnostic chest CT scans were performed on various scanners with 16- to 320- channel multidetector CT (Brilliance 16–64, Philips Medical Systems; SOMATOM Drive/Force/Definition Edge/Definition Flash, Siemens Healthcare; Aquilion ONE, Canon Medical Systems). The scanning parameters were as follow: 0.5-s tube rotation time, collimation:16 $\times$ 75 mm, pitch: 0.938, 120 kVp, 200 mAs with automatic tube current modulation, 1- and 3-mm slice thickness, and post-processing with high-spatial-frequency algorithm. The radiologists analyzed the ILD patterns and included patients who had typical UIP CT patterns (honeycombing and reticular pattern with or without traction bronchiectasis) and probable UIP CT patterns (basal/subpleural predominant reticular pattern and traction bronchiectasis) according to guidelines released in 2018^22,23^. In case of discordance, the same two radiologists had a discussion until they reached a consensus diagnosis. Accordingly, 73 patients were diagnosed with a UIP pattern of ILD and possible malignant lesions. Of these patients, three were excluded from this study (follow-up loss [n = 1], pleural mass [n = 1], and multiple biopsies for multiple lesions on one day [n = 1]). Finally, 70 patients (61 patients with typical UIP pattern and 9 patients with probable UIP pattern) who underwent CT-guided PCNB were included in this study.

### Biopsy protocol

All patients were admitted prior to biopsy and underwent laboratory tests, including measurement of platelet count, prothrombin time, and activated prothrombin time as per the institutional protocol. In cases showing abnormalities, a biopsy was performed after appropriate correction and consultation with a hematologist.

One of three board-certified thoracic radiologists (19, 7, and 3 years of experience) performed CT-guided PCNB as a routine daily procedure. Earlier diagnostic chest CT images were reviewed to determine the optimal biopsy needle path and patient position and predict biopsy-related risks. Before the procedure, written informed consent was obtained from each patient. CT-guided PCNB was performed using a 16- or 320-channel scanner (Brilliance 16, Philips Healthcare or Aquilion ONE, Canon Medical Systems). A preliminary CT scan of the area of interest was taken to locate the puncture site, and the needle trajectory was planned to avoid the ribs and traverse the pleural surface/lung tissue minimally. Local anesthetic (1% lidocaine) was injected subcutaneously at the needle puncture site. The needle was inserted and advanced close to the target lesion. Intermittent CT scans were taken to examine the needle path during the procedure. After reaching the target lesion, specimens were obtained using a 20-gauge coaxial cutting needle system (Mission, Bard). For histopathological examination, specimens were immediately immersed in a 10% formalin solution.

### Post-procedure imaging and patient care

After the biopsy, the patients were observed in the ward and positioned with the puncture side down. Immediate post-procedure CT images were obtained in selected cases according to the operator’s preference. In accordance with the protocol, chest radiography was performed 4 h after the biopsy to rule out complications such as pneumothorax and hemorrhage. In cases showing pneumothorax development, conservative treatment was administered with supplemental oxygen and monitoring of vital signs. In patients who showed signs of respiratory distress or a large amount of pneumothorax, a chest tube was inserted. Patients who did not have complications or had minimal pneumothorax were discharged the following day.

### Data collection

One board-certified radiologist (3 years of experience in chest radiology) collected data from the electronic medical record system and reviewed CT images.

#### Patient and lesion-related information

Patient-related variables such as age, sex, smoking history (never smoker, ex-smoker, or current smoker), and results of the pulmonary function test (forced vital capacity [FVC] and diffusing capacity of the lungs for carbon monoxide [DLCO]) were recorded. The lesion-related information included the location (upper, middle, and/or lower lobe), size (long-axis diameter on axial CT images) and nodule density (solid or subsolid). In addition, the presence of emphysema was evaluated.

#### Procedure-related information

The procedure-related variables included the patient’s position, number of core biopsies, length of the needle path (distance from the pleura to the target), penetration of honeycombing, needle tip within the target, and procedure time. PCNB-related complications were assessed and categorized (minor vs. major) in accordance with the guidelines of the Society of Interventional Radiology^24,25^. Minor complications included pneumothorax, transient hemoptysis, and pulmonary alveolar hemorrhage. Pneumothorax necessitating chest tube insertion, hemoptysis requiring embolization, air embolism, acute exacerbation of ILD, and death were classified as major complications.

#### Pathological results

Original pathological reports were recorded. The PCNB results were categorized as malignant (including atypical adenomatous hyperplasia), benign (specific benign or nonspecific benign), or non-evaluable due to insufficient specimens, while the reference standard results were blinded. Specific benign was defined as a specific benign tumor or infection with an identified pathogen^26^. Nonspecific benign results were defined as the presence of benign features such as inflammatory cells or fibrosis that were insufficient to establish a specific diagnosis^27^. Non-evaluable results were defined as cases in which the obtained specimens were insufficient or inadequate for establishing a pathologic diagnosis.

To identify the proportion of nondiagnostic results, we categorized the PCNB results as diagnostic results (malignancy and specific benign) and nondiagnostic results (nonspecific benign and non-evaluable) according to the categorization described in previous studies^28,29^.

### Reference standards

The reference standard was established as follows^15,30^. First, if the lesion was surgically resected, a surgical pathology report was used to establish the diagnosis. Second, if the biopsy result revealed malignant or specific benign lesions, it was based on pathologic analysis^27^. Third, the lesion was considered benign if it remained stable for at least 2 years for solid nodules and 5 years for subsolid nodule according to the Fleischner Society guideline^31^ or decreased in size with conservative treatment. Fourth, when the clinical behavior revealed an obvious malignant process such as metastasis or rapid tumor progression, the lesion was considered malignant.

### Statistical analysis

The overall diagnostic accuracy, sensitivity, specificity, positive predictive value (PPV), and negative predictive value (NPV) were evaluated. We calculated the overall diagnostic accuracy, including the non-evaluable results. Based on the intention-to-diagnose principle, non-evaluable results were considered false negatives when calculating sensitivity and false positives when calculating specificity^32^.

The proportion of nondiagnostic results was calculated and the overall complication rate was then determined. The risk factors for nondiagnostic results and pneumothorax were identified using Pearson χ^2^ or Fisher’s exact test for categorical variables and unpaired Student’s t-test for continuous variables. A p value < 0.05 was considered statistically significant. Statistical analysis was performed using SPSS version 25.0 software (IBM Corp.).

### Consent for publication

The content has not been published nor submitted for publication elsewhere.

## Results

Of the 70 patients, 61 showed a UIP pattern with honeycombing and 9 showed a probable UIP pattern without honeycombing. Table 1 shows the patient demographics and lesion characteristics. There were 63 men (90.0%) and 7 women (10.0%), with a mean patient age of 73.5 years. Sixteen patients were nonsmokers, 34 were current smokers, and 20 were ex-smokers.

**Table 1:** Demographics of the patients and characteristics of the lesions; *SD* standard deviation.

Characteristic	Value
**Age**	
Median	73.5
Range	58–85
**Sex**	
Male	63
Female	7
**Smoking**	
Smoker	34
Ex-smoker	20
Non-smoker	16
**Location**	
Right upper lobe	12
Right middle lobe	3
Right lower lobe	28
Left upper lobe	12
Left lower lobe	15
**Nodule density**	
Solid	66
Part solid	4
Lesion size (cm) ± SD [range]	4.0 ± 2.2 [1.1 – 11.3]
Length of needle path (pleura to lesion) (cm) ± SD [range]	1.2 ± 1.8 [0.0 – 8.8]
**Forced vital capacity (%)**	n = 63
≥ 90	24
50–89	38
< 50	1
**Diffusing capacity for carbon monoxide (%)**	n = 60
≥ 90	0
50–89	29
< 50	31
**Emphysema**	
Yes	36
No	34

Of the 70 procedures, 53 (75.7%) showed malignant results, 11 (15.7%) showed benign results, and 6 (8.6%) showed non-evaluable results. The final diagnosis was malignant lesions in 62 patients and benign lesions in 8 patients (Table 2). Malignant lesions were confirmed by biopsy or surgical resection (n = 55), biopsy of another organ (n = 3), or post-procedural malignant process (n = 4). A final diagnosis of benign disease was confirmed based on microbiological confirmation (n = 4), decrease of lesion size with conservative treatment (n = 3), and stable lesion size for at least 2 years (n = 1). The overall diagnostic accuracy, sensitivity, specificity, PPV, and NPV for the diagnosis of lung cancer were 85.7% (60/70), 85.5% (53/62), 87.5% (7/8), 100.0% (53/53), and 63.6% (7/11), respectively.

**Table 2:** Reference standard and results of CT-guided percutaneous core needle biopsy.

	Reference standard		Total
	Malignant	Benign	
**Biopsy result**			
Malignant	53	0	53
Benign	4	7	11
Non-evaluable	5	1	6
Total	62	8	70

**Figure 1:**
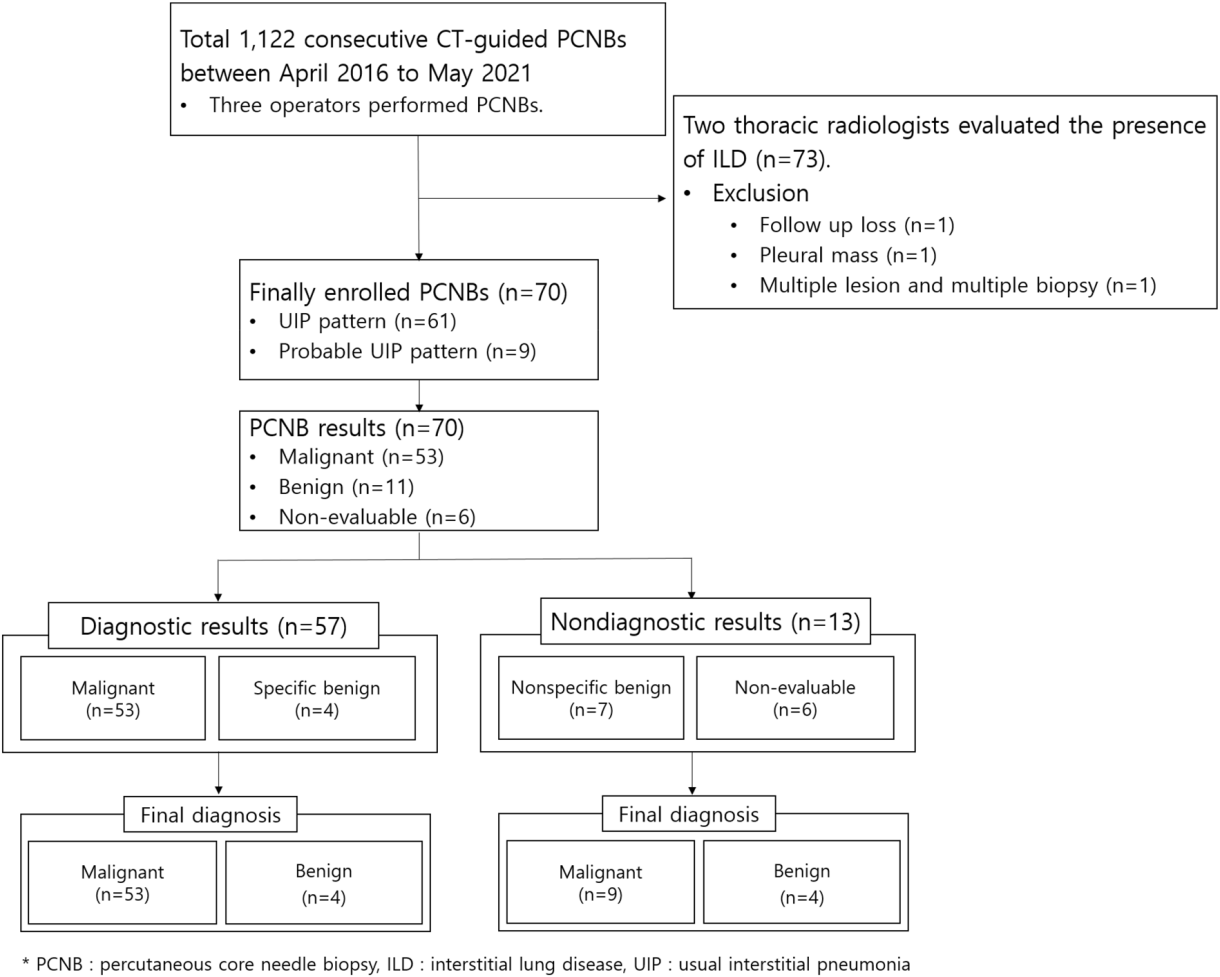
Patient flow diagram and pathology results of percutaneous core needle biopsies (PCNBs) in this study.

The proportion of nondiagnostic results was 18.6% (13/70). The nondiagnostic results consisted of seven nonspecific benign results (three true-negative and four false-negative) and six non-evaluable results (Fig. 1). Table 3 shows the identified risk factors for nondiagnostic results. Two or less core biopsy sampling was related to nondiagnostic results (*p* = 0.002). FVC, DLCO, and penetration of honeycombing were not related to the nondiagnostic results. Furthermore, the presence of the needle tip within the target was not related to the nondiagnostic results in this study (*p* = 0.416) (Table 3).

**Table 3:** Risk factors for nondiagnostic results. *p value of Pearson χ^2^ test or Fisher’s exact test for categorical variables and Student's t-test for continuous variables. *SD* standard deviation.

Factors	Diagnostic result (n = 57)	Nondiagnostic result (n = 13)	*p* value*
Age ± SD	72.9 ± 6.2	71.5 ± 4.7	0.466
**Smoking**			0.201
Smoker	28	6	
Ex-smoker	17	2	
Non-smoker	12	5	
Lesion size (cm) ± SD	4.14 ± 2.14	3.44 ± 2.40	0.302
≤ 2 cm	10	4	0.277
> 2 cm	47	9	
**ILD-type**			0.353
UIP	41	10	
Probable UIP	6	3	
**Forced vital capacity (%)**			0.731
≥ 90	20	4	
50–89	33	5	
< 50	1	0	
**Diffusing capacity for carbon monoxide (%)**			1.000
50–89	25	4	
< 50	26	5	
**Emphysema**			0.365
Yes	31	5	
No	26	8	
Length of needle path (cm) ± SD	0.99 ± 1.34	2.03 ± 2.94	0.236
**Subpleural location (< 2 cm)**			0.455
Yes	46	9	
No	11	4	
**Nodule density**			0.431
Solid	53	13	
Part solid	4	0	
**Position**			0.789
Supine	18	3	
Prone	38	10	
Others	1	0	
**Operator**			0.222
A	5	3	
B	14	4	
C	38	6	
**Number of core biopsy**			**0.003**
1–2	9	7	
≥ 3	48	6	
**Penetration of honeycombing**			0.718
Yes	14	2	
No	43	11	
**Tip within target**			0.416
Yes	49	10	
No	8	3	
Procedure time (min) ± SD	22.06 ± 0.10	25.42 ± 0.07	0.372

Significant values are in bold.

The overall complication rate was 35.7% (25/70). The minor complication rate was 31.4% (22/70), and the major complication rate was 4.3% (3/70). Pneumothorax developed in 22 patients (31.4%), and subsequent chest tube insertion was needed in three patients (4.3%). Pulmonary alveolar hemorrhage without symptoms developed in three patients (4.3%), and mild hemoptysis developed in two patients (2.9%). Neither condition required any intervention. Two patients showed bilateral pneumothorax and pulmonary alveolar hemorrhage. All cases of major complications involved pneumothorax requiring chest tube drainage. No major complications, including PCNB-related air embolism, acute exacerbation of ILD, and death occurred.

**Figure 2:**
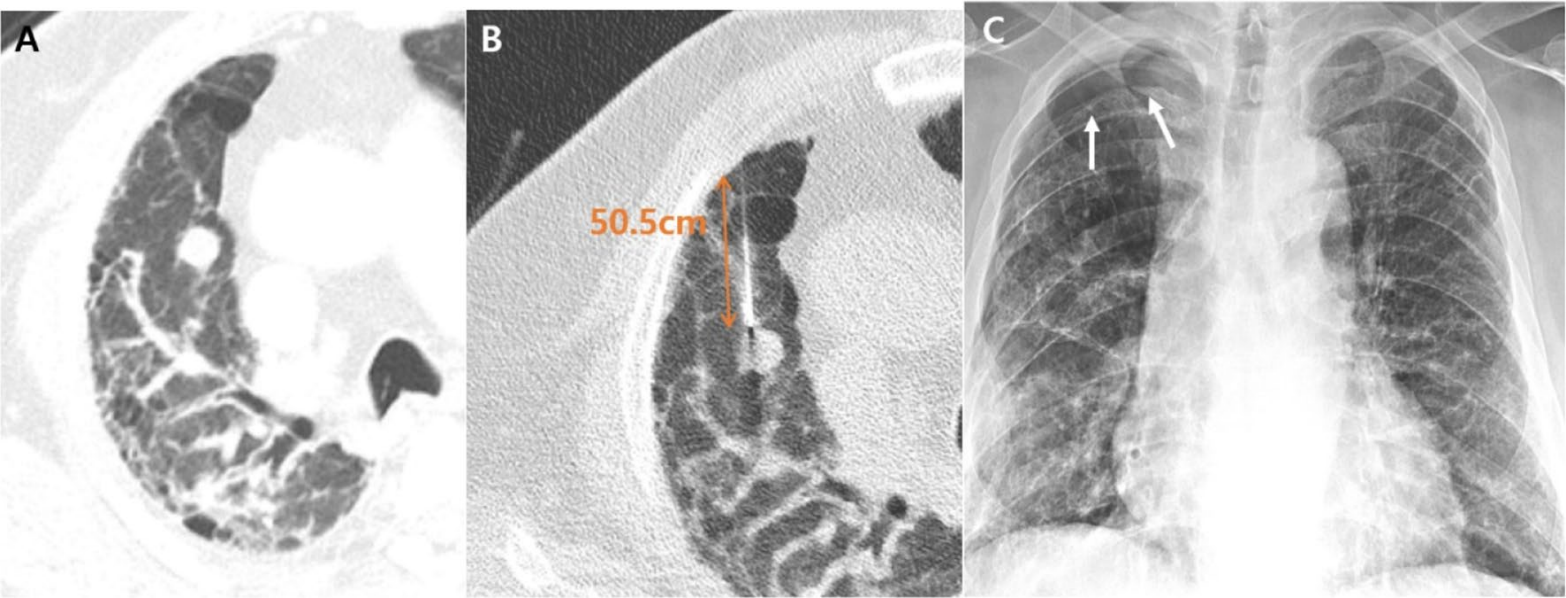
A 69-year-old male patient with idiopathic pulmonary fibrosis. The patient showed a 1.4-cm nodule in his right upper lobe (**A**). CT-guided percutaneous core needle biopsy (PCNB) was performed with the patient in the supine position. The length of the needle path (pleura to lesion) was 5.1 cm (**B**). Small cell carcinoma was confirmed through PCNB. He presented with pneumothorax after PCNB on follow-up radiography (**C**).

**Figure 3:**
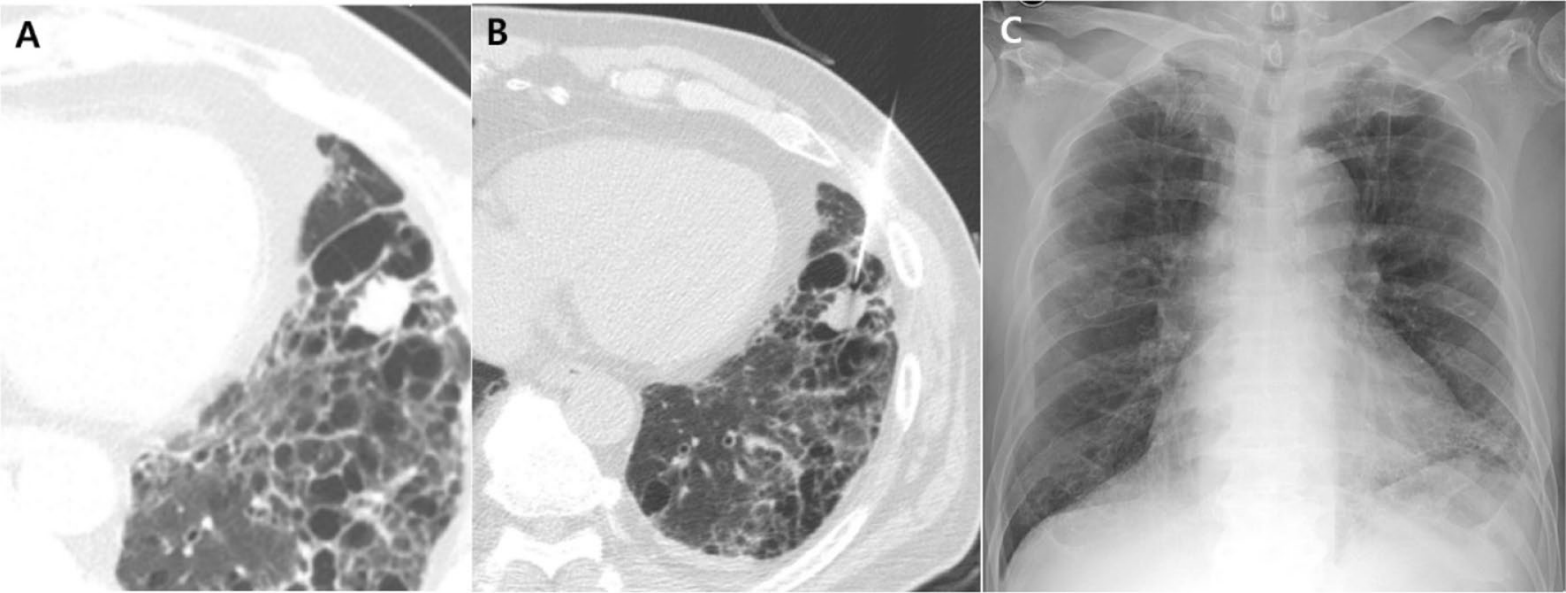
A 67-year-old male patient with underlying idiopathic pulmonary fibrosis. The patient showed a 1.6-cm irregular nodule in his left lower lobe (**A**). CT-guided percutaneous core needle biopsy (PCNB) was performed with the patient in the supine position. The needle traversed the honeycomb cysts (**B**). There was no complication after PCNB (**C**). Squamous cell carcinoma was confirmed through PCNB.

Table 4 shows the identified risk factors for pneumothorax. The needle path was significantly longer in patients with pneumothorax (*p* = 0.007) (Fig. 2, Table 4). Similarly, non-subpleural lesions showed a higher incidence of pneumothorax than subpleural lesions (*p* = 0.002) (Table 4). FVC, DLCO, and penetration of honeycombing were not related to the incidence of pneumothorax (*p* = 0.713, 1.000, and 0.760, respectively) (Fig. 3).

**Table 4:** Risk factors for pneumothorax. *p value of Pearson χ^2^ test or Fisher’s exact test for categorical variables and Student's t-test for continuous variables. *SD* standard deviation.

Factors	Pneumothorax (n = 22)	No pneumothorax (n = 48)	*p* value*
Age ± SD	72.1 ± 5.7	72.9 ± 6.1	0.642
**Smoking**			0.449
Smoker	13	21	
Ex-smoker	4	15	
Non-smoker	5	12	
**Lesion size (cm) ± SD**	3.41 ± 1.98	4.29 ± 2.24	0.119
≤ 2 cm	6	8	
> 2 cm	16	40	
**ILD-type**			0.128
UIP	17	44	
Probable UIP	5	4	
**Forced vital capacity (%)**			0.713
≥ 90	9	15	
50–89	11	27	
< 50	0	1	
**Diffusing capacity for carbon monoxide (%)**			1.000
50–89	9	20	
< 50	9	22	
**Emphysema**			1.000
Yes	11	25	
No	11	23	
Length of needle path ± SD	2.18 ± 2.16	0.73 ± 1.35	**0.007**
**Subpleural location (< 2 cm)**			**0.002**
Yes	12	43	
No	10	5	
**Nodule density**			0.585
Solid	20	46	
Part solid	2	2	
**Position**			0.190
Supine	8	13	
Prone	13	35	
Others	1	0	
**Operator**			0.061
A	5	3	
B	3	15	
C	14	30	
**Number of core biopsy**			0.986
1–2	5	11	
≥ 3	17	37	
**Penetration of honeycombing**			0.760
Yes	6	10	
No	16	38	
**Tip within target**			0.303
Yes	17	42	
No	5	6	
Procedure time (min) ± SD	24.51 ± 10.32	21.15 ± 10.32	0.339

Significant values are in bold.

## Discussion

We assessed the diagnostic performance and complication rate of CT-guided PCNB by using a coaxial needle in patients with a UIP pattern of ILD and evaluated the risk factors for nondiagnostic results and pneumothorax. Although PCNB has been established as a safe diagnostic procedure, only one study has evaluated the diagnostic accuracy of percutaneous lung biopsy in patients with UIP^17^. That study used both fine-needle aspiration and core biopsy results and included only a few cases using a coaxial needle. To our knowledge, this is the first study to analyze the diagnostic accuracy and safety of CT-guided PCNB with a coaxial needle in patients with UIP.

We found that the overall diagnostic accuracy, sensitivity, and specificity of CT-guided PCNB with a coaxial needle in patients with UIP were 85.7%, 85.5%, and 87.5%, respectively. These results are slightly lower than those reported in previous studies^11,30,33,34^. However, those studies excluded non-evaluable results for calculating diagnostic accuracy, sensitivity, and specificity. As we considered non-evaluable results as false-negative or false-positive while calculating diagnostic accuracy, sensitivity, and specificity, the diagnostic value was lower than that reported in the aforementioned studies. Furthermore, the reduction in diagnostic accuracy was relatively large due to the small number of cases.

Shin et al. reported that CT-guided lung biopsy had an 89% diagnostic accuracy in patients with a UIP/IPF pattern^17^; our result is slightly lower, but comparable. They reported that the presence of the needle tip outside the target and a small lesion size were risk factors for nondiagnostic results. In contrast to their findings, these factors were not significantly related to the nondiagnostic results in our study. Our study revealed that the availability of three or more core biopsy samples significantly increased the diagnostic results. This may be due to the reduced sampling error. These findings are consistent with those of previous studies using a coaxial needle system, which documented that the availability of three or more specimens was associated with a higher diagnostic accuracy^15,35^. Pulmonary function (FVC and DLCO) was not related to nondiagnostic results.

The overall complication rate and major complication rate were 35.7% (25/70) and 4.3% (3/70), respectively. These rates were much lower than those reported in a previous study that reported an overall complication rate of 51% and a major complication rate of 38% in patients with a UIP/IPF pattern^17^. However, the two studies had many differences in patient group and procedure technique, so an exact comparison of both studies is difficult. However, we conjectured that one of the possible causes of the difference in complication rates is the use of coaxial needles. The coaxial technique allows for much easier sampling repetition without increasing the number of pleural passages^19,20^. In theory, the complication rate is thought to be low because of the smaller pleural injury as a result of avoiding repeated pleural punctures. However, a comparative study between the coaxial needle group and the non-coaxial needle group in patients with a UIP pattern of ILD is needed for verification.

Pneumothorax is the most common complication of CT-guided PCNB, and the reported incidence after lung biopsy ranges between 17 and 60%^10,36–38^. In our study, pneumothorax occurred in 22 of 70 patients (31.4%). This rate was within the range of values reported in previous studies. In our study, a higher rate of pneumothorax was associated with a deeper location of the lesion from the pleural surface. Several studies reported comparable results to ours^12,37,39–41^. It would be reasonable to assume that the longer the needle remains in the lung parenchyma, the greater the risk of tearing the pleura as the patient breathes during the procedure^37,42^. Furthermore, needle redirection may be more frequently required with a longer needle path, which could result in pleural injury^39^. In contrast, Yeow et al.^43^ reported that subpleural location was a risk factor for pneumothorax because insufficient anchoring caused the needle to dislodge into the pleural space easily, resulting in air ingress. However, in our study, non-subpleural lesions presented with a higher incidence of pneumothorax than subpleural lesions.

Pneumothorax itself is an occasional complication of UIP^16,44^. Therefore, we predicted that patients with UIP would show a higher incidence of pneumothorax after PCNB. However, the incidence was within a reasonable range in comparison with previous studies that were not limited to patients with UIP^10,36–38^. Shin et al. reported that penetration of honeycomb cysts was a risk factor for major complications after lung biopsy in patients with a UIP/IPF pattern^17^. However, the penetration of honeycomb cysts was not associated with pneumothorax or complications in our study.

This study had several limitations. First, this study was a retrospective study that included a small sample. The retrospective design of this study might have resulted in selection bias. In addition, owing to the small sample size, some risk factors for nondiagnostic results and pneumothorax might have failed to exhibit statistical significance. Second, the differences in diagnostic accuracy and complication rates between the groups with and without UIP were not compared. Third, we included patients with a UIP pattern ILD based on CT findings using updated guidelines^22,23^. The UIP pattern is observed not only in IPF but also in collagen vascular disease associated ILD, hypersensitivity pneumonitis, and asbestosis. Since it includes heterogeneous disease entities, the clinical characteristics of each disease cannot be reflected. Fourth, at the time of biopsy, the majority of patients in our study had relatively good pulmonary function. It is possible that only patients who were considered clinically capable of PCNB were included, implying that our study cohort might not be representative of all patients with UIP. Finally, the number of occasions in which the pleural surface was repeatedly penetrated was not recorded in detail, although it was very low.

In conclusion, CT-guided PCNB with a coaxial cutting needle is a useful diagnostic technique for the diagnosis of lung cancer, even in patients with UIP. It yields reasonable diagnostic accuracy and an acceptable complication rate.

## Data Availability

The datasets generated during and/or analyzed during the current study are available from the corresponding author on reasonable request.

## References

[1] Lynch DA (2005). Idiopathic interstitial pneumonias: CT features. Radiology.

[2] Adegunsoye A (2019). Computed tomography honeycombing identifies a progressive fibrotic phenotype with increased mortality across diverse interstitial lung diseases. Ann. Am. Thorac. Soc..

[3] Salisbury ML (2019). Hypersensitivity pneumonitis: Radiologic phenotypes are associated with distinct survival time and pulmonary function trajectory. Chest.

[4] Zamora-Legoff JA, Krause ML, Crowson CS, Ryu JH, Matteson EL (2017). Progressive decline of lung function in rheumatoid arthritis-associated interstitial lung disease. Arthrit. Rheumatol..

[5] Walsh SL (2014). Connective tissue disease related fibrotic lung disease: high resolution computed tomographic and pulmonary function indices as prognostic determinants. Thorax.

[6] Le Jeune I (2007). The incidence of cancer in patients with idiopathic pulmonary fibrosis and sarcoidosis in the UK. Respir. Med..

[7] Naccache JM (2018). Lung cancer and interstitial lung disease: A literature review. J. Thorac. Dis..

[8] Xiaohong X (2021). Management and prognosis of interstitial lung disease with lung cancer (ILD-LC): A real-world cohort from three medical centers in China. Front. Mol. Biosci..

[9] Lee KH (2019). Diagnostic accuracy of percutaneous transthoracic needle lung biopsies: a multicenter study. Korean J. Radiol..

[10] Yoon SH (2019). Analysis of complications of percutaneous transthoracic needle biopsy using CT-Guidance modalities in a multicenter cohort of 10568 biopsies. Korean J. Radiol..

[11] Kim TJ (2008). Diagnostic accuracy of CT-guided core biopsy of ground-glass opacity pulmonary lesions. AJR Am. J. Roentgenol..

[12] Laurent, F., Michel, P., Latrabe, V., Tunon de Lara, M. & Marthan, R. Pneumothoraces and chest tube placement after CT-guided transthoracic lung biopsy using a coaxial technique: Incidence and risk factors. *AJR Am. J. Roentgenol.* **172**, 1049–105310.2214/ajr.172.4.10587145 10.2214/ajr.172.4.10587145 10587145

[13] Laurent F, Montaudon M, Latrabe V, Begueret H (2003). Percutaneous biopsy in lung cancer. Eur. J. Radiol..

[14] Manhire A (2003). Guidelines for radiologically guided lung biopsy. Thorax.

[15] Hiraki T (2009). CT fluoroscopy-guided biopsy of 1,000 pulmonary lesions performed with 20-gauge coaxial cutting needles: Diagnostic yield and risk factors for diagnostic failure. Chest.

[16] Yamazaki R (2021). Pneumothorax in patients with idiopathic pulmonary fibrosis: A real-world experience. BMC Pulm. Med..

[17] Shin YJ (2021). Accuracy and complications of percutaneous transthoracic needle lung biopsy for the diagnosis of malignancy in patients with idiopathic pulmonary fibrosis. Eur. Radiol..

[18] Laurent F, Latrabe V, Vergier B, Michel P (2000). Percutaneous CT-guided biopsy of the lung: Comparison between aspiration and automated cutting needles using a coaxial technique. Cardiovasc. Interv. Radiol..

[19] Zhang L (2018). Coaxial technique-promoted diagnostic accuracy of CT-guided percutaneous cutting needle biopsy for small and deep lung lesions. PLoS ONE.

[20] Nour-Eldin NE (2016). Pneumothorax Complicating Coaxial and Non-coaxial CT-Guided Lung Biopsy: Comparative analysis of determining risk factors and management of pneumothorax in a retrospective review of 650 patients. Cardiovasc. Interv. Radiol..

[21] Yoon SH (2021). 2020 Clinical Practice Guideline for Percutaneous Transthoracic Needle Biopsy of Pulmonary Lesions: A consensus statement and recommendations of the korean society of thoracic radiology. Korean J. Radiol..

[22] Raghu G (2018). Diagnosis of Idiopathic Pulmonary Fibrosis. An official ATS/ERS/JRS/ALAT clinical practice guideline. Am. J. Respir. Crit. Care Med..

[23] Lynch DA (2018). Diagnostic criteria for idiopathic pulmonary fibrosis: A Fleischner Society White Paper. Lancet Respir. Med..

[24] Veltri A, Bargellini I, Giorgi L, Almeida P, Akhan O (2017). CIRSE guidelines on percutaneous needle biopsy (PNB). Cardiovasc. Interv. Radiol..

[25] Sacks D, McClenny TE, Cardella JF, Lewis CA (2003). Society of interventional radiology clinical practice guidelines. J. Vasc. Interv. Radiol..

[26] Gelbman BD (2012). Radiographic and clinical characterization of false negative results from CT-guided needle biopsies of lung nodules. J. Thorac. Oncol..

[27] Kim JI, Park CM, Kim H, Lee JH, Goo JM (2017). Non-specific benign pathological results on transthoracic core-needle biopsy: how to differentiate false-negatives?. Eur. Radiol..

[28] Haas BM (2016). Nondiagnostic computed tomography-guided percutaneous lung biopsies are more likely when infection is suspected. J. Thorac. Imag..

[29] Lee KH (2019). Nondiagnostic percutaneous transthoracic needle biopsy of lung lesions: A multicenter study of malignancy risk. Radiology.

[30] Lee SM (2014). C-arm cone-beam CT-guided percutaneous transthoracic needle biopsy of lung nodules: Clinical experience in 1108 patients. Radiology.

[31] MacMahon H (2017). Guidelines for management of incidental pulmonary nodules detected on CT images: From the Fleischner Society 2017. Radiology.

[32] Schuetz GM, Schlattmann P, Dewey M (2012). Use of 3x2 tables with an intention to diagnose approach to assess clinical performance of diagnostic tests: Meta-analytical evaluation of coronary CT angiography studies. BMJ.

[33] Choi SH (2013). Percutaneous CT-guided aspiration and core biopsy of pulmonary nodules smaller than 1 cm: Analysis of outcomes of 305 procedures from a tertiary referral center. AJR Am. J. Roentgenol..

[34] Hur J (2009). Diagnostic accuracy of CT fluoroscopy-guided needle aspiration biopsy of ground-glass opacity pulmonary lesions. AJR Am. J. Roentgenol..

[35] Lim C, Lee KY, Kim YK, Ko JM, Han DH (2013). CT-guided core biopsy of malignant lung lesions: How many needle passes are needed?. J. Med. Imag. Radiat. Oncol..

[36] Otto S (2015). Predictors of technical success and rate of complications of image-guided percutaneous transthoracic lung needle biopsy of pulmonary tumors. PLoS ONE.

[37] Khan MF (2008). Variables affecting the risk of pneumothorax and intrapulmonal hemorrhage in CT-guided transthoracic biopsy. Eur. Radiol..

[38] Maxwell AW (2014). CT-guided transthoracic needle aspiration biopsy of subsolid lung lesions. J. Vasc. Interv. Radiol..

[39] Cox JE, Chiles C, McManus CM, Aquino SL, Choplin RH (1999). Transthoracic needle aspiration biopsy: Variables that affect risk of pneumothorax. Radiology.

[40] Topal U, Ediz B (2003). Transthoracic needle biopsy: Factors effecting risk of pneumothorax. Eur. J. Radiol..

[41] Chami HA (2015). Predictors of pneumothorax after CT-guided transthoracic needle lung biopsy: The role of quantitative CT. Clin. Radiol..

[42] Kinoshita F (2006). CT-guided transthoracic needle biopsy using a puncture site-down positioning technique. AJR Am. J. Roentgenol..

[43] Yeow KM (2001). Risk factors for pneumothorax and bleeding after CT-guided percutaneous coaxial cutting needle biopsy of lung lesions. J. Vasc. Interv. Radiol..

[44] Franquet T, Gimenez A, Torrubia S, Sabate JM, Rodriguez-Arias JM (2000). Spontaneous pneumothorax and pneumomediastinum in IPF. Eur. Radiol..

